# Evaluation of the “Let’s Get Organized” group intervention to improve time management: protocol for a multi-centre randomised controlled trial

**DOI:** 10.1186/s13063-021-05578-x

**Published:** 2021-09-19

**Authors:** Marie Holmefur, Afsaneh Roshanay, Suzanne White, Gunnel Janeslätt, Elin Vimefall, Kajsa Lidström-Holmqvist

**Affiliations:** 1 School of Health Sciences, Faculty of Medicine and Health, Örebro University, Örebro, Sweden; 2 Department of Public Health and Caring Sciences, Uppsala University, Uppsala, Sweden; 3 State University of New York, Downstate Medical Center, Brooklyn, New York, USA; 4 Department of Public Health and Caring Sciences, Uppsala University and Centre for Clinical Research in Dalarna, Uppsala University, Falun, Sweden; 5 Örebro University School of Business, Faculty of Business, Science and Engineering, Örebro University, Örebro, Sweden; 6 University Health Care Research Center, Faculty of Medicine and Health, Örebro University, Örebro, Sweden

**Keywords:** Time management skills, Mental disorders, Neurodevelopmental disorders, Intervention, Self-efficacy, Occupational balance, Cost-effectiveness

## Abstract

**Background:**

Time management skills are essential for living in modern society. People with mental or neurodevelopmental disorders typically have cognitive limitations, including affected time management, which might lead to poor occupational balance, low self-efficacy, and poor parental sense of competence. “Let’s Get Organized” (LGO) is a recently developed manual-based group intervention to train time management skills. The aim of this trial is to evaluate the efficiency of the Swedish version of LGO (LGO-S) compared to treatment as usual (individual occupational therapy) to improve time management for adults with impaired time management skills due to mental or neurodevelopmental disorders. Furthermore, to evaluate if the intervention is a cost-effective way to improve the quality of life and time management skills of these individuals, we will conduct a health economic evaluation.

**Methods:**

The trial will have a multi-centre, open, parallel randomised controlled design. A total of 104 adults with cognitive limitations due to mental or neurodevelopmental disorders will be recruited from open psychiatric or habilitation care units. Outcomes will be measured before and after a 10-week intervention, with a follow-up 3 months after completing the intervention. The primary outcome will be self-assessed time management skills. Secondary outcomes will be e.g. self-assessed skills in organisation and planning, regulation of emotions, satisfaction with daily occupations, occupational balance, self-efficacy, and quality-adjusted life years.

**Discussion:**

A recent feasibility study has shown promising results for LGO-S, and a randomised trial will provide robust evidence for the possible efficacy of LGO-S in comparison to treatment as usual.

**Trial registration:**

ClinicalTrials.gov NCT03654248. Registered on 20 August 2018.

**Supplementary Information:**

The online version contains supplementary material available at 10.1186/s13063-021-05578-x.

## Background

In today’s time-dependent society, time management is a fundamental skill that is essential for carrying out daily routines and undertaking single and multiple tasks independently and with others [1]. It is necessary to establish or maintain occupational balance, and it is a crucial requirement for living a satisfying life and for maintaining employment in modern society [2]. Research has shown that in adults, time management skills are positively associated with perceived job satisfaction and health and negatively associated with stress [3, 4]. Difficulties with time management are frequently reported in persons with diagnoses such as mental or neurodevelopmental disorders, and methods to improve and compensate for these difficulties are needed. This paper reports on a planned trial to evaluate the efficacy of a time management intervention called “Let’s Get Organized” for adults with cognitive limitations due to mental or neurodevelopmental disorders.

### Defining time management and closely related constructs

*Time management* includes the mental functions of ordering events in chronological sequence and allocating amounts of time to events and activities [1]. In the literature and in this trial, *time management skills* refers to both the cognitive function described above [1] and the skill to manage complex behaviours to make effective use of time in goal-directed activities within the time allotted in daily life [3]. Time management is one of several components included in executive functions.

Executive functions are a multifaceted neuropsychological construct defined by the World Health Organization (WHO) as specific mental functions that are especially dependent on the frontal lobes of the brain, including complex, goal-directed behaviours such as decision-making, abstract thinking, planning and carrying out plans, mental flexibility, and deciding which behaviours are appropriate under the given circumstances [1]. There is general agreement that there are three main executive functions, namely inhibitory control (including self-control), working memory, and cognitive flexibility [5–7]. From these skills, higher-level cognitive functions such as reasoning, problem solving, and planning are built [8, 9]. Time management is included in these complex behaviours, as well as the closely connected functions of organisation and planning [1].

*Organisation and planning* are the cognitive functions of coordinating parts into a whole and of systematising the mental function involved in developing a method of proceeding or acting [1]. In the literature, the acronym OTMP (organisation, time management, and planning) is frequently used as a term to mirror the interrelated nature of organisation, time management, and planning [10, 11]. To illustrate the difference between time management and organisational skills, Solanto et al. [12] divide the different therapeutic targets and component skills into three parts—time management (including time estimation), effective use of planners, and when and for how long to complete tasks—while organisational skills refer to what to do and where to do it and planning includes goal-setting and prioritising.

### Time management difficulties for people with mental or neurodevelopmental disorders

People with mental and neurodevelopmental disorders rate their time management skills significantly lower than people without cognitive disabilities [13]. Despite different causal pathways for executive dysfunction between these diagnostic groups, the consequences in daily life in terms of time management are not that different. For people with attention-deficit/hyperactivity disorder (ADHD), difficulties in time management in daily life are commonly reported [14–16], and these have been explained as the convergence of impairments in time perception, attention, impulse control, and planning [17]. People with autism spectrum disorder (ASD) have a more generalised impairment of all aspects of executive functioning, but share similar difficulties in handling time in daily life [18, 19]. Furthermore, research has pointed to impaired executive functioning in various mental disorders, such as depression or schizophrenia [20, 21], leading to difficulties in organising the activities of daily life. Among people with serious mental illness, time use is commonly restricted to eating, caring for oneself, and performing quiet activities or sleeping [22]. This pattern of restricted time use significantly reduces community participation, even after taking employment status into account [23, 24].

### Consequences of, or issues related to, time management difficulties

Experiences of failure in time management are associated with difficulties in a number of other areas of life. This trial will put the spotlight on occupational balance, regulation of emotions, general self-efficacy, and sense of competence in a parental role. Human occupation has been described in a number of conceptual models as the interplay between the occupation (or activity), the person, and the context [25]. Occupations should be balanced in order to either establish or maintain health. *Occupational balance* is defined as organising daily activities to allow for variation between different types of activities in labour and work, home management, parenting, leisure, and rest activities [26]. In order to establish and maintain occupational balance, time management skills and planning and organisation abilities are important. All activities need to be planned and carried out, taking time into account, which affects the occupational balance over the day, week, and month. Claessens et al. [3] state that time management and organisation skills are essential for maintaining occupational balance, which is crucial for health and well-being and can reduce stress. Occupational imbalance can manifest itself in activity deprivation, which means having only a few recurring daily activities in a limited number of settings [22], thus leading to poor satisfaction with daily life [23]. In others, the imbalance could manifest in far too many activities in a variety of settings, thus leading to extensive problems in managing time, as is often described in persons with ADHD as a result of difficulties in estimating and planning time [15].

Symptoms of inefficient time management, including difficulties in finishing tasks on time and procrastination [27], in people with neurodevelopmental disorders might, beyond executive dysfunction, be related to difficulties in *regulation of emotions* [28]. Emotion regulation involves changes in emotion dynamics and is defined as the process by which we control which emotions we have and how we experience their magnitude, intensity, and duration [29] and how we express them [1, 30]. Emotion regulation changes in structure during the life span from mostly behavioural strategies in childhood to more cognitive strategies in adulthood [31].

Furthermore, for a person to gain control over their life, a sense of self-efficacy is important. *Self-efficacy* is defined as the beliefs in one’s capacity to complete a task, organise and perform the courses of action required to produce a given accomplishment [32, 33], and it has shown to be related to time management skills [34]. It is assumed that self-efficacy affects the individual’s behaviour, such as the tasks and approaches they choose, as well as their efforts and performances.

Expanding the general conceptualisation of self-efficacy [32, 35, 36] to the specific case of being a parent results in describing parental self-efficacy, which is the parents’ beliefs about how well they fulfil their parental role, i.e. their ability to parent effectively and influence the behaviour and environment of their children in a way that would benefit their children’s development and success. Parental self-efficacy is the theoretically central cognitive component of the *parent’s sense of competence* [32, 37, 38]. A recent study showed that general self-efficacy is associated with time management skills in parents without disabilities [13], and parents with disabilities rate their general self-efficacy and parental sense of competence as lower than parents without disabilities [13]. Regulation of emotion, mentioned earlier, is also important for parenting [39, 40]. Emotion regulation difficulties in parents with ADHD can make it difficult for them to manage their affects and to react consistently and calmly to their children [41]. It has also been shown that regulation of emotions in parents with cognitive disabilities is related to the parental sense of competence, both in efficacy and in satisfaction [13]. In parents with cognitive disabilities, the satisfaction in parental sense of competence is significantly associated with time management, organisation, and regulation of emotions [13]. Due to the negative impact of impaired time management skills in daily life, there is a great need for cost-effective interventions to improve time management.

### Interventions to improve time management

A number of different interventions to improve time management in daily life have been investigated in the literature. Most attention in research has been given to children with ADHD, and less to adults and persons with other reasons for impaired time management skills.

Adult clients with time management difficulties within mental health settings typically meet an occupational therapist for individual assessments and interventions targeting daily structure and training in the use of cognitive assistive technology. Cognitive assistive technology can help adults with ADHD in managing everyday life [42], but such technology alone has only moderate evidence to support improved time management [43]. Interviews with people with cognitive disabilities using time-assistive technology have indicated that electronic planning devices are perceived as helpful and can aid in organisation, managing time, and improving volition [44]. However, these products alone are usually not sufficient to enable the person to function fully in daily life [45]. For adults with ADHD, there is some evidence that systematic meta-cognitive therapy can enhance time management and organisational skills [12, 46]. Furthermore, an individual intervention focusing on time-use to improve occupational balance and engagement has shown clinical usefulness in people with serious mental disorders [47].

In recent years, structured group interventions have emerged, aiming to improve occupational balance and prevent occupational deprivation in people with mental disorders. One such method is “Balancing everyday life”, which showed good results in one study [48]. Nonetheless, there is currently no documented evidence-based intervention aiming to improve time management and organisational skills in adults with mental or neurodevelopmental disorders. A promising method that focuses specifically on time management skills is the “Let’s Get Organized” (LGO) intervention [49, 50]. The goal of LGO is to foster the development of effective time management habits and organisational skills in clinical settings. In LGO, time management skills include an awareness that time can be managed effectively through the active use of skills, strategies, and tools, and the intervention helps the individual to create and maintain a flexible routine while exercising emotional control [51]. LGO encompasses the core components that were identified by Langberg et al. as necessary for developing time management skills [46]. The learning principles are trial-and-error learning strategies, task analysis, task sequencing, and behavioural strategies [49, 50]. Cognitive assistive techniques, such as maintaining an appointment book and using goal-directed strategies for cognitive rehabilitation, are employed [49, 51]. The original LGO intervention was clinically evaluated with people with serious mental disorders and substance-related disorders in a small (*n* = 16), pre-post study in the USA and showed significant improvement in time management skills [49].

The LGO intervention has been translated and adapted to a Swedish context and is called LGO-S [52]. LGO-S is intended for use in clinical settings for people with neurodevelopmental or mental disorders or mild intellectual disability [53]. A feasibility study of LGO-S was performed in Swedish open psychiatric clinics with 75 participants with mental and/or neurodevelopmental disorders (dropouts *n* = 20) [52]. The results showed significant improvements in time management, organisation and planning, and regulation of emotions. These improvements were retained in both the 3-month and 12-month follow-ups [52, 54]. Furthermore, the number of activities the participants performed in their daily life and their satisfaction with these activities improved significantly during the study, as well as some aspects of executive functioning [52]. A qualitative interview study with participants in the LGO-S intervention rendered rich descriptions of how they had managed to increase the structure in their daily life and to accomplish more tasks. The participants also indicated an increase in self-efficacy in relation to planning and time management and the experienced impact of belonging to a group [55]. In order to establish to what extent the improvements highlighted in the feasibility study are due to LGO-S and have an effect over and above usual treatment, a randomised controlled trial is needed.

## Methods: participants, interventions, and outcomes

### Objectives

The aim of this trial is to evaluate the effectiveness of the LGO-S intervention in open psychiatric or habilitation care to improve time management for adults with impaired time management skills due to mental or neurodevelopmental disorders.

In comparison to treatment as usual (TAU, namely individual occupational therapy), we hypothesise that LGO-S is:

- More effective in improving self-assessed time management skills, regulation of emotions, satisfaction with daily occupations, occupational balance, and self-efficacy
- Equally effective in improving self-assessed organisation and planning skills, aspects of executive functioning, and psychiatric symptoms
- More effective in improving parental sense of competence for parents with children living at home
- A cost-effective method to increase the individual’s quality of life and self-reported time management skills

### Trial design

The trial will employ a multi-centre, open, parallel, two-armed randomised controlled design to compare LGO-S with TAU. The allocation ratio is 1:1. The trial design was informed by the Consolidated Standards of Reporting Trials guidelines [56].

#### Trial setting

The trial will be conducted in ten units in Örebro, Dalarna, Uppsala and Stockholm counties, including seven open psychiatric care units , one community care unit for young adults in upper secondary school with a diagnosed or suspected neurodevelopmental disorder (ADHD or ASD), and two open habilitation care units for adults with ASD; see Additional file 1 for trial centres.

#### Eligibility criteria

Eligible people will be adults (age 18–65 years) with self-reported problems with time management in daily life and a diagnosed mental and/or neurodevelopmental disorder or who are undergoing medical investigation for a neurodevelopmental disorder. Further inclusion criteria are that possible pharmacological treatment is stable and that the person can complete the Weekly Calendar Planning Activity test (regardless of the result). The four exclusion criteria for prospective participants are a diagnosed intellectual disability, the inability to communicate in Swedish, if they have previously taken part in the LGO-S intervention, or if they are currently undergoing any other lifestyle intervention.

### Interventions

Two interventions will be compared in this trial. They are the LGO-S group intervention and TAU, which is individual occupational therapy.

#### The “Let’s Get Organized” group intervention

LGO-S is a 16-week group intervention with weekly 1.5-h sessions and is divided into two parts. Part 1 (10 sessions) is focused on time management, and part 2 (6 sessions) is focused on organising and planning activities [57]. In this trial, only part 1 will be evaluated. Each group has six to eight participants and is led by two trained group leaders. The key mechanisms in the LGO-S intervention are goal-directed and other learning strategies, which are used to train effective time management habits such as maintaining a calendar and wearing a watch. The LGO-S material is comprised of a course manual for the leaders with accompanying PowerPoint presentations and working material for each session. White [49] (p 713) asserts that group sessions follow the same structure with specific stages, “to ensure integration and generalisation of new learning and establish proper habit formation”. Each group session has a set theme, which is different for each session (Table 1), and six stages that are common to all ten sessions:

- In stage 1, clients sign the attendance sheets and indicate the time of their arrival. Then, they identify and record their current emotional states on a sheet of paper that is placed in their personal folders.
- In stage 2, in the first session, appointment books are distributed if the participants do not have their own. At each subsequent session, participants review and enter new information into their appointment books, sharing various ways of personalising them through the use of colour coding, family photographs, and Post-It notes. Habit-building experiences and resistance to using the appointment book are discussed.
- In stage 3, the theme of the day (Table 1) is presented in a PowerPoint presentation. Activity worksheets relating to time management and organisation from Precin’s *Living Skills for Recovery Workbook* [58] will be used with modifications of the activities to conform to Allen’s Level 5, meaning that overt trial-and-error learning is facilitated [59].
- In stage 4, discussion of the completed worksheet or activity reassures participants that using trial-and-error to correct mistakes is an acceptable and valuable learning tool. The group norm—that “mistakes are OK”—is intended to promote respect for each other’s efforts, lower participants’ performance anxiety, and encourage willingness to try out new behaviours. Clients are encouraged to notice their own and others’ learning styles.
- In stage 5, participants are given homework. The homework requires them to use their appointment books daily and to be cognisant of time management strategies learned in LGO-S sessions.
- In stage 6, participants clean up materials from the session and place their worksheet in their personal folder. Participants are informed of the next group topic, which is intended to create anticipation and motivation for future participation. The group ends with a few minutes of calm and an expression of thanks for the group session.

**Table 1:** LGO-S intervention, content of sessions

Session 1	Appointment books Define trial-and-error learning Identifying and managing time strengths and weaknesses
Session 2	Appointment books Managing time strengths and weaknesses How to make time work for me: case study 1
Session 3	Appointment books Barriers for managing time in activities What I dislike or have to do versus what I like to do
Session 4	Appointment books Using an activity schedule to get an overview of time and routines: case study 2 Estimating and measuring the duration of time in daily activities (homework)
Session 5	Appointment books How do I spend my time? Using an activity schedule to be in control of time Prioritising time using a to-do list (homework)
Session 6	Appointment books Making the most of my time and energy Energy levels and circadian rhythms
Session 7	Appointment books Making the most of my time and energy—your activities and the energy needed for them Measuring attention span (homework)
Session 8	Appointment books Revising my schedule, daily routines, and improving routines. Practice altering one routine (homework)
Session 9	Appointment books Time to have fun—weekend planning. Rewarding myself
Session 10	Appointment books What have I learned. Evaluation of the LGO. Diploma

The outline and essential components of the programme have been further described by White [49]. The cultural adaptations in LGO-S are described in Holmefur et al. [52].

The leaders of the LGO-S intervention are required to attend a 2-day training in the LGO-S intervention method. In addition, at least one of the group leaders for each group needs to be an occupational therapist (OT). The providers of individual occupational therapy are required to be OTs, but they are not required to have completed the LGO training. In this paper, both the group leaders and the providers of individual occupational therapy are referred to as interventionists.

#### Individual occupational therapy (TAU)

The control intervention will be TAU, which is individual occupational therapy services targeting time management, organisation, and planning. This intervention involves both individual coaching and training, as well as prescriptions of time-assistive products. Sweden’s National Board of Health and Welfare’s mandatory web education called “The Prescription Process” guides the process of product selection, when necessary adaptations are made, information, education and training, follow-up, and evaluation [60]. The TAU intervention does not necessarily standardise the number of visits or the duration of the programme. Rather, each intervention is individually tailored. Thus, the OTs are instructed to perform the intervention as they would usually do, which includes one or more visits for assessments followed by advice regarding structuring of activities in daily life, devising schedules or other low-tech aids for time management, prescription or advice on time-assistive devices, and conducting training in how to use time-assistive devices in daily life. The TAU in this trial will be limited to 10 weeks.

#### Participant timeline

**Fig. 1:**
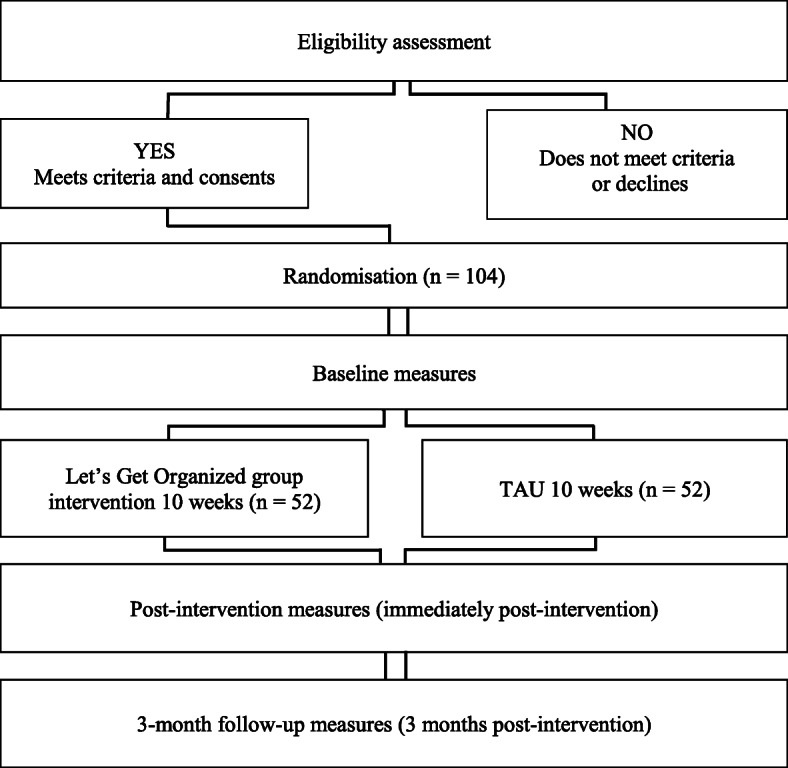
Flow of participants through the trial

Figure 1 describes the flow of potential participants in the trial.

### Outcomes

All outcomes will be measured at 1–2 weeks prior to the intervention, 1–2 weeks after the intervention, and 3 months after the intervention has concluded (Table 2). The primary endpoint used will be immediately (1–2 weeks) after the completed intervention.

**Table 2:**
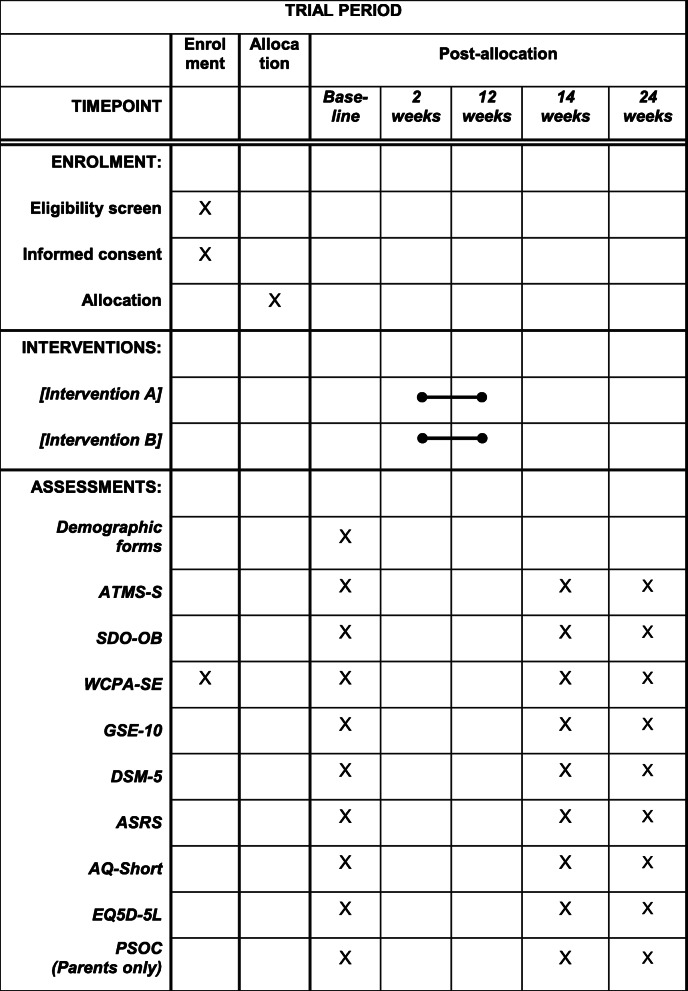
Participant timeline. Time schedule of enrolment, interventions, and assessments of participants

*ATMS-S* Assessment of Time Management Skills – Swedish version, *SDO-OB* Satisfaction with Daily Occupation – Occupational Balance, *WCPA-SE* Weekly Calendar Planning Activity – Swedish version, *GSE-10* General Self Efficacy Scale 10 items, *DSM-5* The DSM-5 Self-Rated Level 1 Cross-Cutting Symptom Measure-Adult, *ASRS* ADHD Self-report Screening scale, *AQ-short* Adult Autism Spectrum Quotient – short form, *PSOC* Parental Sense of Competence

#### Assessment of Time Management Skills (ATMS-S)

The primary outcome of this trial will be the domain of self-reported time management skills as measured by the Swedish version of the Assessment of Time Management Skills (ATMS-S) questionnaire. The ATMS-S is a self-administered questionnaire with 27 items. Each item is to be answered on a 4-point rating scale. The ATMS-S measures three constructs. The items are thus divided into three subscales, including time management (11 items, the primary outcome), organisation and planning (11 items), and regulation of emotions (5 items). Raw scores from each subscale are transformed into ATMS units, which is a Rasch-based continuous measure ranging from 0 to 100, where a higher value represents better skills [61]. The specific metric used will be change from baseline to completed intervention as the primary point of comparison, and as a secondary comparison change from baseline to 3 months after the completed intervention. The mean/median values will be reported and analysed. The test–retest reliability has been evaluated with good results (the whole scale in the American version had an *r* = 0.89, and for each subscale in the Swedish version, the intraclass correlation coefficient ranged from 0.82 to 0.86) [51, 62].

#### Satisfaction with Daily Occupations – Occupational Balance (SDO-OB)

Occupational balance and satisfaction with daily occupations will be measured by the interview-based questionnaire Satisfaction with Daily Occupations – Occupational Balance (SDO-OB) and measured as change from baseline [63, 64]. The SDO-OB contains 13 items aiming to estimate a person’s satisfaction with their daily activities in the four activity areas of work (3 items), leisure (3 items), domestic tasks (4 items), and self-care occupations (3 items). For each item, the person is asked to answer whether the activity was performed during a given time interval (yes or no). The scores are summed on an activity scale ranging from 0 to 13 activities and data are continuous and will be reported as mean and SD. The level of satisfaction with the performance of each activity is rated on a satisfaction scale from 1 (extremely dissatisfied) to 7 (extremely satisfied). The sum score of satisfaction has a total range of 13 to 91; data are continuous and reported as mean and SD. For each activity area, the participants are asked to rate their perceived occupational balance on a 5-grade scale, ranging from 2 (far too much) to −2 (far too little), which are considered as categorical data and will not be summed but regarded as profiles of occupational balance. These profiles will be categorised and proportions of balance profiles will be compared. Furthermore, the SDO-OB is concluded with two questions regarding global satisfaction with daily occupations and global occupational balance. Global satisfaction with daily occupations is rated from 1 to 5, where 1 indicates the best possible satisfaction with daily occupations. Global occupational balance is rated from 1 to 5, where 1 is far too much engagement in activities and 5 is far too little. Both global scales will be reported as mean and SD in change from baseline. The construct validity of the SDO-OB has been established [64]. The SDO activity and satisfaction scales with 13 items have been shown to have good psychometric properties indicating internal consistency (*α* = 0.83)[65], good validity [63, 65], and good test–retest reliability with *r*_s_ = 0.84 (satisfaction) and *r*_s_ = 0.92 (activity) [66].

#### Weekly Calendar Planning Activity (WCPA-SE)

Executive functioning will be measured with the Swedish version of the Weekly Calendar Planning Activity (WCPA-SE) [67]. The WCPA-SE measures a person’s ability to coordinate and integrate different aspects of executive functioning, such as organisation and planning, inhibition of distractions, monitoring the passage of time, and strategies used in a cognitively challenging planning task [68]. When administering the WCPA-SE, the person is given a list of 17 appointments and meetings to be entered into a blank weekly calendar. The test is timed, with five rules to adhere to, and the strategies used by the person are noted by the test leader. The WCPA-SE has three difficulty levels. In this trial, level 2 will be used. A recent study has shown that the test–retest reliability between two tests was weak, but when repeated, the reliability between tests two and three was acceptable to excellent (ICC = 0.65–0.91) [69]. Thus, to reduce random variation and possible learning effects, two alternative lists of appointments will be used along with two baseline tests, first at enrolment and then at the time of the other baseline assessments. The second assessment will be used as the baseline assessment [69]. The outcomes used in this trial will be the total number of correctly entered appointments, the total time to complete the task, the number of rules followed, the number of strategies used, and the efficiency score [67]. All these outcomes are continuous data and will be reported as mean (SD) as change from baseline.

#### General Self-Efficacy Scale (GSE-10)

Self-efficacy will be measured with the Swedish version of the General Self Efficacy Scale (GSES-10). The GSES-10 has 10 items that are scored on a 4-point rating scale. The GSES-10 measures self-rated belief in personal competence to effectively deal with a range of stressful situations and is reported with a continuous score range of 10–40. Higher scores indicate better self-efficacy and will be reported as mean (SD). The GSE-10 has been translated into 26 languages and has been used internationally and has shown good validity and reliability [70].

#### Symptom scales

Three scales will be used to monitor psychiatric symptoms. The first scale will be the DSM-5 Self-Rated Level 1 Cross-Cutting Symptom Measure-Adult in Swedish, with 23 items in 13 domains of mental health. Participants report a self-rated measure of recent symptoms rated from none (0) to severe (4) [71]. Scores will be summed by domain and presented as mean (SD). The second scale used will be the World Health Organization adult ADHD self-report screening scale (ASRS). This is an 18-item ADHD symptom questionnaire scored on a 5-point rating scale from never to very often and intended for use in the general population [72]. The first six items have shown to be predictive of ADHD and will be summed and reported as continuous data using mean (SD). The third scale used will be the Swedish version of the Adult Autism Spectrum Quotient (AQ-short), which is used to monitor autism symptoms. This scale includes 28 statements with four possible responses from definitely disagree (1) to definitely agree (4) [73] and will be scored according to a specific scoring key provided by the instrument developer. Scores will be reported as continuous and as mean (SD).

#### Cost-effectiveness measure

To enable a cost-utility analysis, we will measure health-related quality of life using the EQ-5D-5L, developed by EuroQol [74]. In the EQ-5D-5L, the individual’s health status is measured in the five dimensions of mobility, self-care, usual activities, pain/discomfort, and anxiety/depression using a 5-level scale ranging from no problem to extreme problems. By using a value set, this can be converted to a health index ranging from 0 (dead) to 1 (perfect health). A Swedish value set is currently under development and if available will be used when analysing the data. By multiplying the change in health-related quality of life by the duration of this change, expressed in years, the result will be the change in quality-adjusted life years (QALYs), which is the outcome most commonly used in health economic evaluations.

#### Parental Sense of Competence

Participants with children living at home will be administered the Parental Sense of Competence Scale (PSOC) to measure the domain of self-efficacy in the parental role. The PSOC is a self-rated questionnaire with 15 items rated on a 6-point rating scale from 1 (strongly agree) to 6 (strongly disagree) that measures parents’ perceptions of their parental skills [75]. The PSOC, used in the general population, measures three factors—satisfaction, efficacy, and interest [76], and as sum will be calculated for each factor which will be reported as continuous and mean (SD). The PSOC has previously been used in assessing parents with ADHD [77] and people with mental and neurodevelopmental disabilities [13].

#### Trial-specific forms

Two trial-specific demographic forms will be collected only at pre-intervention. One form will be filled out by the participant with their information, including age, sex, family status, living arrangements, and information on education and work. The second form will be filled out by the recruiting staff and contains information on diagnosis, medication, length of contact with the services, and any prior prescribed time-assistive devices and contacts with an occupational therapist. In the case of dropouts, the time of occurrence will be noted, along with the reason if given by the participant.

To evaluate the process of implementing the interventions, a form will be filled out for each participant by the interventionist. For participants receiving the LGO-S intervention, the form will document the number of sessions, as well as which sessions they attended. For participants receiving TAU, the form will document the number of sessions, the time duration per session, the content of any interventions, and prescribed and/or recommended cognitive assistive devices.

### Sample size

A total of 104 participants will be recruited for the trial. There will be 52 participants in each group. The sample size calculation is based on the earlier feasibility study showing that an increase of 8 ATMS units in mean difference (SD = 10 for both groups) on the time management subscale of the ATMS-S was considered a significant effect [52]. Furthermore, the calculation took into account an 80% power to detect change, a dropout rate of 25%, and a statistical significance level of *p* < 0.01.

### Recruitment

The goal is to recruit approximately 13 participants from each participating unit. Participants will be recruited by treating OTs or special education teachers from patient rosters in the units. The eligibility assessment, including administration of the WCPA-SE, will be done by the recruiting staff. If the person is found eligible, oral and written information will be given about the trial, including the two possible interventions and the randomisation process. If they agree to participate, a consent form will be signed by the participant.

## Methods: assignment of interventions

### Allocation (randomisation)

#### Sequence generation

The randomised allocation will be performed by using a computer-generated numbering sequence which will be carried out by a professional academic statistician. The basic requirement is a ratio of 1:1. A multiple varying block randomisation stratified by centre will be performed. The reason for this strategy is to allow for differing numbers of recruited participants from different centres.

#### Allocation concealment mechanism

The allocation sequence and block randomisation strategy will be concealed from the staff recruiting the participants. Each interventionist will be given a personal code and will assign the recruited participant a unique participant number consisting of the interventionist’s personal code with an added serial number. For each new participant, the recruiting staff will contact the trial coordinator via telephone or e-mail, who will reveal the allocated intervention for the specific participant.

#### Blinding (masking)

Blinding to intervention allocation of interventionists or participants will not be possible in this trial. The interventionists conducting TAU will however be blinded to baseline assessment results and the statistician will be blinded to group allocation.

## Methods: data collection, management, and analysis

### Data collection methods

Data for participants in the LGO-S intervention will be collected by trained group leaders who will be local OTs working at the participating clinics. Along with the mentioned training workshop in the LGO-S intervention, all group leaders will also be trained in administering the measures used in this trial. During training, the group leaders will be instructed to ensure that data is complete at the time of data collection while being with the participant. As stated above, data for participants in the TAU intervention will be collected by the research group. For each participant, the same data collector will collect all data in order to avoid inter-rater bias, as far as practically possible.

### Data management

To manage the data, a web-based system called Smart-Trail (S-T) will be used. S-T is a secure system and the content is tailored for this trial with web-based forms for each data collection point. There is automatic marking for the event that data are missing or entered inaccurately. Each participant can be efficiently followed throughout the trial, and the data are organised by unit. The system thus gives a clear visual overview of each participant’s status. The use of S-T fulfils the requirements of the European General Data Protection Regulation (GDPR). Only parts of the research group will have access to S-T. To gain access to the system, the principal investigator has to approve of this action, and also decide what role the researcher who is gaining access will have (i.e. entering or viewing data). The S-T has a two-step login function and each login is tracked by the system.

Directly after randomisation, the participant will be registered in S-T with their participant code and intervention allocation. Raw data will then be collected on paper forms, one bundle for each data collection point containing all measures applicable to the specific occasion. The forms in the bundles are in their original formats and have not been changed by the research group. Forms will be marked with the participant’s code and entered manually by the researchers. When the data collection is finished and all raw data are entered into S-T, the data will be exported to statistical software for further analyses.

### Statistical analysis plan

#### General statistical considerations

The flow of participants through the study will be illustrated according to the Consolidation Standard of Reporting Trials (CONSORT, Fig. 1) [78].

Prior to data analysis, raw data from each instrument will be coded according to the instructions for the respective instrument. Statistical tests will be two-sided and conducted at the conventional (two-sided) 5% level with no formal adjustment for multiple testing. Secondary outcomes are likely to be highly correlated so that standard adjustment techniques, such as the Bonferroni method, would be conservative [79].

All outcome measures and patient characteristics will be summarised by their trial allocation group and by measurement point. The distribution of outcome data will be examined. The main analysis of outcome data will be the comparison of change from baseline between treatment arms. Change from baseline has a “normalising” effect and standard techniques are generally applicable. To maintain the baseline comparability of the compared groups, the main analyses will be performed on an intention-to-treat basis, meaning that participants will be analysed in the arm they were allocated to. A researcher blinded to group allocation will conduct the statistical analysis, guided by a professional academic statistician (also blinded).

#### Analysis of baseline data

Baseline demographic and clinical characteristics will be reported for each treatment arm using descriptive statistics (mean and SD or median and IQR for continuous variables, and absolute number and percentage for categorical variables). Normality will be assessed graphically and with the Shapiro–Wilk test.

#### Primary outcome analysis

The pre-specified primary endpoint in this trial is the change from baseline in time management skills as measured with the ATMS-S immediately after the intervention compared between the LGO-S and TAU treatment arms. Differences will be analysed with an independent *t*-test or the Mann–Whitney *U*-test, depending on the distribution.

#### Sensitivity analyses for the primary outcome

Sensitivity analyses will be performed to assess the impact of areas of uncertainty surrounding the primary outcome analysis and its robustness. The data within centres may be correlated, and a regression analysis appropriate for the data distributions and model assumptions will be conducted, which will allow for this clustering in order to obtain an unbiased estimation of the treatment effect and its standard error (SE). Other factors that could threaten the robustness of results might be (1) how the outcome is defined, i.e. if time management skills is expressed as a continuous or a dichotomous variable; (2) number of sessions attended in the LGO-S; and (3) differences in characteristics between participants lost to follow-up and those staying in the trial. Analyses related to point 2 above will involve conducting a per-protocol analysis in which individuals having participated in 7 or more sessions are regarded as “programme completers” (i.e. as having complied with the protocol sufficiently).

#### Secondary outcome analysis

Secondary outcomes will be analysed according to the same procedure as the primary outcome analysis. If applicable, a secondary analysis for best responders in the trial will be conducted by pooling the two treatment arms and applying a suitable regression model.

#### Handling of missing data

Missing data may compromise findings from randomised clinical trials, particularly if missingness is not at random (MAR) and if missing data are not handled appropriately [80]. We will follow recommendations in the paper by Jakobsen et al. to handle missing data. This may include best-worst and worst-best sensitivity analyses, and full information maximum likelihood [80]. In the case of single missing observations on the three subscales of ATMS-S, person measures (subscale units per person) will be calculated using anchored item difficulty and category structure in a Rasch analysis. Winsteps software (updated version) will be used for Rasch analyses.

### Health economic evaluation

The health economic evaluation will be based on a societal perspective and use the change in QALYs as the main outcome variable. Based on the change in QALYs, the cost per QALY will be calculated by the incremental cost-effectiveness ratio (ICER) as follows:

$$ \mathrm{ICER}=\frac{C_i-{C}_j}{e_i-{e}_j}, $$

where *c*_*i*_ and *e*_*i*_ are the cost and effect for the intervention group and *c*_*j*_ and *e*_*j*_ are the cost and effect for the control group. The cost included here is the cost for the interventions (LGO-S and TAU), which will be estimated based on the information provided by the interventionist regarding the activities that are performed and the time that it takes to perform them. The cost measure will include both direct costs, such as the materials that are used, and time-use costs. For LGO-S, this will also include the cost of the 2-day training that the interventionists are required to attend. There will be no access to other health or social care costs.

Apart from calculating the ICER based on the QALYs gained, we will also incorporate a cost-effectiveness analysis using self-reported time management skills as measured with the ATMS-S as the main outcome.

## Methods: monitoring

### Data monitoring

Staff that are providing intervention and collecting parts of the data have been trained in giving the LGO-S intervention by the Swedish Association of Occupational Therapists. To maintain adherence to the trial protocol, interventionists will be gathered for network meetings four times a year, and this will be complemented with visits to the units by the researchers. There is a risk that participants will discontinue treatment or be partially absent from treatment in either treatment arm due to e.g. lack of motivation, lack of time, worsened health or meeting, and travel restrictions due to the COVID-19 pandemic. All deviations from the protocol will be noted by the interventionists. Interim analyses will be performed when 50% of the participants have completed their last assessment. The data collection will stop when the stipulated number of participants is reached or when the principal investigator makes such a decision based on interim analyses. The principal investigator will have the final decision regarding the termination of the trial. No stopping guidelines have been deemed necessary because there are no, or very minor, risks with participation. The extended research group (the authors of this protocol, and in addition two other colleagues) functions as a steering committee that the PI and the project coordinator will report to on a regular basis and also discuss issues with arising during the trial.

## Ethics and dissemination

Ethical permission to conduct this trial was granted by the Regional Ethical Review Board in Uppsala, Sweden (Dnr: 2018/191). Any important protocol modifications will be reported to the Board for approval and will be registered in ClinicalTrials.gov. Informed written consent will be obtained after verbal and written information by the recruiting staff at the participating units. The information letter informs about the aim and nature of the study, the rationale for the study, possible harms and benefits from participating in the study, how data is handled, how results can be accessed, that participation is voluntary, and that participants can withdraw at any time without giving reason. Informed consent documents can be obtained from the first author. Sensitive personal data regarding health state will be collected in this trial. To secure confidentiality, each participant will have a code that will be used with all data that are collected, and the only links between the codes and the participants’ names will be in a code list, which will be stored locally and securely at the School of Health Sciences at Örebro University along with, but apart from, the written consent forms and the raw data.

### Declaration of interests

The investigators have no financial or competing interests pertaining to this trial.

### Ancillary and post-trial care

Participants who are allocated to TAU will be offered to participate in the LGO-S intervention after completing all assessments in this trial. Likewise, individual TAU will be offered to participants in the LGO-S intervention. Participants will be monitored for psychological well-being throughout the trial by the interventionists. If needed, psychological support from the treating unit will be offered to the participant.

### Dissemination policy

The results of this trial will be reported on a group level in the form of publications in scientific journals and presentations at conferences, and they will also be reported to the participants and participating units in the form of lectures and reports. The researchers will seek to gain public interest for the trial results.

## Discussion

As shown in the literature review, difficulties with time management are frequently reported in people with diagnoses such as mental or neurodevelopmental disorders. The consequences of such difficulties for these people and for society as a whole speak for a need for methods to overcome these difficulties. There are, to our knowledge, currently no documented evidence-based interventions aiming to improve time management and organisational skills in adults with mental or neurodevelopmental disorders. The trial described in this paper will create new evidence for the efficacy of the LGO-S intervention, which is designed to target time management skills. The earlier results from the feasibility study of the LGO-S are promising and warrant the need for a randomised trial, which will provide robust evidence regarding its efficiency and whether there are any improvements beyond those of TAU. The qualitative study of participants in LGO-S suggested that the group setting format further enhances the experience of self-efficacy after the intervention, but whether this is true or not remains to be seen in this trial.

The results of the trial will inform us further on the possible benefits of LGO-S compared to TAU in improving self-assessed time management skills, regulation of emotions, satisfaction with daily occupations, occupational balance, and self-efficacy. It will also increase our understanding as to whether LGO-S is equally efficient as TAU at improving self-assessed organisation and planning skills, improving aspects of executive functioning, and improving psychiatric symptoms. Furthermore, this trial will add knowledge about whether LGO-S is more efficient than TAU in improving parental sense of competence for parents with children living at home. Finally, the trial will demonstrate whether LGO-S is a cost-effective way to target time management skills in the clinical setting.

When including an intervention such as TAU in a study, one foreseen challenge is the individual intervention design, involving both strategy building and prescription of time-assistive devices. While there is research evaluating time-assistive devices, the whole intervention package with all its variations has not yet been studied. Nevertheless, this is the nature, and the beauty, of clinical occupational therapy, and as such, it needs to be included in trials. In this trial, we will need to compensate for the vague and general description of TAU beforehand by providing more detailed descriptions of the method when reporting on the trial results. A limitation of this trial is the lack of blinding to group allocation by assessors and interventionists. The outcomes in this trial are however mostly self-report measures and they are therefore more prone to participant bias and only secondarily to assessor bias. Participant bias is subject to potential response shift, i.e. that increased awareness will make the participants more strict in their self-assessment after intervention than before. In that case, the effect of intervention would be underestimated. This possible response shift could however occur in both treatment arms and the effect on between-group differences might be less affected. Another limitation in this trial is the lack of public involvement in the design, planning, and execution of the trial. The health economic evaluation will be limited by the lack of access to general health and social care costs over and above the costs of interventions.

This trial has the advantage of being carried out in close collaboration with the clinics in the trial, and the implementation of the intervention is included in the trial design. This means that if LGO-S is proven to be an effective intervention, there are forms for implementation in place for further use in clinical settings.

## Trial status

Recruitment started in August 2018.

Currently recruiting (75 out of 104 as of April 9, 2021) and completion planned by December 2022.

Protocol version 3, 28 August 2021

## Supplementary Information

**Additional file 1.** Trial centres.

## Data Availability

Not applicable

## Abbreviations

AQ-short: Adult Autism Spectrum Quotient – short form
ASRS: ADHD Self-report Screening scale
ATMS-S: Assessment of Time Management Skills – Swedish version
DSM-5: The DSM-5 Self-Rated Level 1 Cross-Cutting Symptom Measure-Adult
GSE-10: General Self Efficacy Scale 10 items
LGO: Let’s Get Organized
LGO-S: Let’s Get Organized Swedish version
PSOC: Parental Sense of Competence
SDO-OB: Satisfaction with Daily Occupation – Occupational Balance
WCPA-SE: Weekly Calendar Planning Activity – Swedish version
