# Supplementary material for: Evaluation of the “Let’s Get Organized” group intervention to improve time management: protocol for a multi-centre randomised controlled trial
Source: Trials. 2021 Sep 19;22:640. doi: 10.1186/s13063-021-05578-x (PMC8449991; doi:10.1186/s13063-021-05578-x)
Supplement: Supplementary file 1 — Additional file 1. Trial centres. [file 13063_2021_5578_MOESM1_ESM.docx]

**Appendix 1: Trial centres**

| Centre | Organization | City |
| --- | --- | --- |
| USÖ psykiatrisk öppenvård och affektiva enheten | Region Örebro County | Örebro, Sweden |
| Karlskoga psykiatrisk öppenvård | Region Örebro County | Karlskoga, Sweden |
| Hallsberg psykiatrisk öppenvård | Region Örebro County | Hallsberg, Sweden |
| Lindesberg psykiatrisk öppenvård | Region Örebro County | Lindesberg, Sweden |
| Vuxenhabiliteringen, autismteamet | Region Örebro County | Örebro, Sweden |
| Socialpsykiatrin | Örebro kommun | Örebro, Sweden |
| NP Resurs och samverkan | Örebro kommun | Örebro, Sweden |
| Öppenvårdspsykiatrisk mottagning | Region Dalarna | Falun, Sweden |
| Neuropsykiatriska mottagningen, Akademiska sjukhuset | Region Uppsala | Uppsala, Sweden |
| Habiliteringscenter Brommaplan vuxna, Habilitering och hälsa | Region Stockholm | Stockholm, Sweden |
